# Exhaustive Variant Interaction Analysis using Multifactor Dimensionality Reduction

**DOI:** 10.21203/rs.3.rs-3401025/v1

**Published:** 2023-10-16

**Authors:** Gonzalo Gómez-Sánchez, Lorena Alonso, Miguel Ángel Pérez, Ignasi Morán, David Torrents, Josep Ll. Berral

**Affiliations:** 1 Barcelona Supercomputing Center (BSC), Barcelona, Spain; 2 Universitat Politècnica de Catalunya - BarcelonaTECH, Barcelona, Spain; 3 Institut Català de Recerca i Estudis Avançats, Barcelona, Spain

## Abstract

One of the main goals of human genetics is to understand the connections between genomic variation and the predisposition to develop a complex disorder. These disease-variant associations are usually studied in a single independent manner, disregarding the possible effect derived from the interaction between genomic variants. In particular, in a background of complex diseases, these interactions can be directly linked to the disorder and may play an important role in disease development. Although their study has been suggested to help to complete the understanding of the genetic bases of complex diseases, this still represents a big challenge due to large computing demands. Here, we have taken advantage of High-Performance Computing technologies to tackle this problem using a combination of machine learning methods and statistical approaches. As a result, we have created a containerized framework that uses Multifactor Dimensionality Reduction to detect pairs of variants associated with Type 2 Diabetes (T2D). This methodology has been tested in the Northwestern University NUgene project cohort using a dataset of 1,883,192 variant pairs with a certain degree of association with T2D. Out of the pairs studied, we have identified 104 significant pairs, two of which exhibit a potential functional relationship with T2D.

## Introduction

The human genome reference sequence is composed of a chain of more than 3 billion nucleotide pairs. By comparing the DNA sequences between any two individuals, up to 3.78 million differences can be found, which are known as genomic variants. In fact, more than 400 million genomic variants have been characterized^1^. Among others, the study of genomic variation is crucial to understand disease predisposition and, therefore, one of the main goals of the Computational Genomics field is to identify disease-associated variants. Particularly, complex diseases, such as Type 2 Diabetes, asthma, or Alzheimer’s disease, are caused by the simultaneous effect of multiple genomic variants and environmental factors.

The necessity for understanding genetically complex diseases has driven the search for significant associations between genomic variants and complex diseases. During the last two decades, these associations have been broadly characterized by Genome-Wide Association Studies (GWAS)^2^. These studies, which rely on a wide variety of methods such as logistic regression or genetic programming^3^, focus on discovering variants associated with the risk of developing the disease. While GWAS performs association tests for each variant in a single independent manner, in variant interaction studies, the search is broadened to inspect the effects from the interaction of variants, which can be both additive or epistatic^4^. To tackle variant interactions studies, different methods, tools, and strategies have then arisen, focusing mainly on pairwise interactions^5^.

There is a wide range of tools and frameworks which can be used to analyze variant interactions. Particularly, some of the tools commonly used for GWAS detection have been adapted to analyze interactions, such as PLINK^6^, which is based on logistic regression, and other frameworks such as GWIS^7^, based on receiver operating characteristic (ROC) curves, or BOOST^8^, which relies on contingency tables and Chi-Square tests, but other techniques have been newly generated to approach the problem. However, despite all these tools facilitating the discovery of variant interactions associated with the disease and the inspection of their effect, the obstacle of analyzing variant interactions is still a computational and methodological challenge: in the simplest approach, where only pairwise interactions are analyzed, the number of combinations to study ascends to more than 10^12^. As a result, the combination of the computational challenge that represents the execution of billions of tests and the extremely restrictive multiple testing burden penalty^9^ required in this type of analysis represent the main limitations for these methods.

One of the potential strategies to overcome these major limitations is a two-stage approach, where the first step involves reducing the dimensionality of the problem. This dimensionality reduction focuses on reducing the number of variant interactions that will be analyzed and in most cases involves filtering the data. This filter can be done, among others, based on data mining techniques, such as the ones used in Relief^10^, where a proximity measure is calculated between individuals on the basis of genome-wide similarity, or using data-integration techniques, which involve selecting only the genomic variants that are relevant to the study based on previous biological information, such as protein-protein interaction databases like ChEMBL^11^ or BioGRID^12^. After reducing the size of the problem, non-exhaustive explorations can be performed using statistical methods and artificial intelligence. In this direction, multiple approaches have been tested and have identified pairwise combinations that show association with complex diseases in reduced datasets. Some examples are random forest^13^, bayesian networks^14^, computational evolution systems^15^, and ant colony optimization^16^.

Importantly, among these methods, Multifactor Dimensionality Reduction (MDR), which is a supervised classification approach, based on contingency tables, has been previously used to approach the analyses of GENE-GENE interactions^17^ and variant-variant interactions with reduced datasets^18^.

In this work, we have developed a containerized framework using High-Performance Computing (HPC) technologies and applied MDR to discover relevant variant interactions in an efficient parallel environment. This method has been tested to find associations with Type 2 Diabetes (T2D) in a subset from the Northwestern University NUgene cohort^19^.

### Contributions:

- Contribution 1: a containerized HPC architecture for detecting disease-associated variant pairs.
- Contribution 2: discovery and functional interpretation of pairs of variant interactions associated with T2D.

From the 1,883,192 pairs studied, 104 were found by the MDR associated with Type 2 Diabetes. From this subset, we have identified two variant pairs where the connection between Type 2 Diabetes can be explained by gene expression genomic variation and variant impact on gene pathways.

## Methods

### Input dataset preparation

The 70KforT2D is a Type 2 Diabetes (T2D) case-control dataset which includes different cohorts with individuals of European ancestry^20^. From this dataset, we analyzed the Northwestern University NUgene project (NUgene) cohort^19^, which consists of 1,128 individuals, 601 non-diabetic and 527 diabetic. The human datasets used for this study are publicly available through the dbGaP platform (phs000237.v1.p1). The access to this data was approved by the Institutional Review Board of the Barcelona Supercomputing Center (BSC). Informed consent was obtained from all subjects to use of their coded DNA samples and data for a broad range of genetic research by third-party investigators.

### Multifactor Dimensionality Reduction

**Figure 1.**
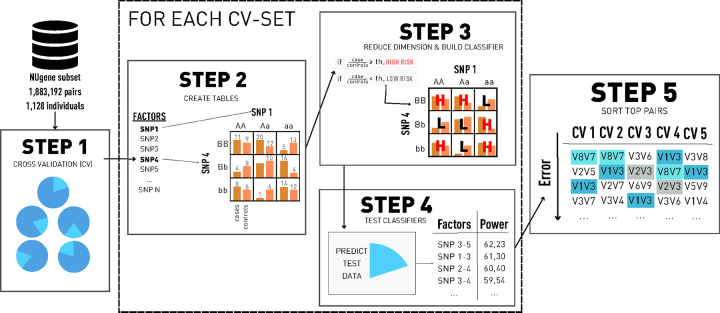
The five steps of the Multifactor Dimensionality Reduction (MDR). In step 1 we build the Cross-Validation (CV) sets. In step 2, for each possible pair of Single Nucleotide Polimorphisms (SNP) in the dataset we create a contingency table. Then, in step 3, we transform each contingency table into a classifier, and, in step 4, we get the prediction power of each pair. Finally, in step 5, we sort the top pairs by their prediction power for each CV set.

MDR is a non-parametric statistical method for detecting and characterizing nonlinear interactions. It follows a naive Bayes approach, building a probabilistic classifier for each variant-variant interaction and finding the best combinations in terms of prediction. The higher the prediction power of a pair is, the more associated with the disease will be. The steps of the method can be found in Figure 1 and are described as follows.

1. The first step consists of a *k*-fold cross-validation and is performed to avoid potential over-fitting. Thus, to find pairs of variants that can have an effect in any dataset, not just the one being evaluated. We divide the data into *k* different sets: then, for each distribution, we determine one of the blocks as the testing set and the others as the training set. Since we generate *k* different distributions of the dataset, the following steps are going to be performed *k* times, one for each distribution.
2. In the second step, we build a contingency table for each variant pair evaluated, only using the individuals from the training dataset. This corresponds with a 2 factors table (variant-variant), each one with 3 classes corresponding to the variant genotypes (AA, Aa, aa), ending with a 9 cells table where each cell is divided into cases and controls.
3. In step three, each of the tables is going to be transformed into a 1-dimension space using the case-control ratio from the training distribution set as a threshold, *T*. The cells where the ratio of cases to controls is greater than *T* are going to be classified as ‘high risk’ and, otherwise, as ‘low risk’. As a result of this step, we reduce the dimension of the problem from 2 classes to 1.
4. In step four, each of the variant-variant tables is used to classify the individuals of the testing set. The classification is done as follows: for each individual, we extract the variant-variant genotypes and check to which class of the multifactor table belongs. Then, if the class corresponds to a high-risk cell, we classify the individual as a case. Otherwise, we classify it as a control. After classifying every individual, we compare the predicted classes with the original labels, obtaining the prediction power of the variant-variant table.
5. After repeating steps two, three, and four for each of the possible cross-validation sets from step one, in step five, the best and most consistent variant-variant combinations are selected. This means picking the ones that appear most times in the cross-validation sets as a top predictor pair.

### Chi-square *p*-value threshold selection

The required *p*-value threshold has been chosen in concordance with the reduction of the dataset for performing MDR in a feasible time and retaining the majority of related pairs to the disease.

### Framework

The optimization of the method is key to make it feasible to analyze current genomic datasets with billions of pairwise combinations or even trios and more complex combinations. We have used High-Performance Computing (HPC)^21^ methodologies and environments to maximize the efficiency of the method, relying on technologies such as Apache Spark^22^ and HDFS^23^. To obtain the best performance using these technologies, first, it is necessary to optimize the method and then, to parallelize it.

The optimization of the method relies on efficient vector multiplications. We use Python^24^ as the main programming language and numpy^25^, a library specially prepared to perform fast vector operations. MDR benefits from the use of this library because it is composed of count and compare actions that can be better defined as matrix multiplications using binarized data, reducing the computational time drastically.

One of the reasons that convert our combined architecture into a great tool for analyzing variant-variant interactions is its scalability capabilities. Since each of the combinations is computed in solitude, with no need to share any information but the final results, it presents an excellent opportunity for parallel computing. We have implemented our methodology using Apache Spark, a tool based on the use of Resilient Distributed Datasets (RDD)^26^. As a result of using Apache Spark, the operations over a data item can be distributed over a cluster of machines, processing the data efficiently in memory. This overcomes the limitations of other technologies in the cluster computing paradigm, such as MapReduce^27^. To maintain track of the operations performed to the RDD in the different nodes, it uses the Directed Acyclic Graph (DAG)^28^.

In order to make these on-memory operations more efficient, it is important to count with a distributed file system that allows fast access to the data in every machine. The use of Hadoop Distributed File System (HDFS)^23^ allows us to distribute the storage, and, by combining it with Apache Spark RDD, we can distribute both the input data and the operations across a cluster system.

Cluster computing and cloud computing is a key resource for HPC applications. In order to increase the compatibility of our architecture, we have developed a singularity container with all the necessary executables, libraries, configuration files, and scripts needed to run the application. The use of containers provides a lightweight and portable solution with less overhead than other solutions, such as virtual machines^29^, increasing the efficiency of our application. We have tested all our frameworks using a cloud-based OpenStack^30^ environment and the Supercomputer MareNostrum IV^31^.

### Functional interpretation analyses

To validate the biological relevance of our results, we conducted a series of functional interpretation analyses. For this, we annotated the resulting loci (LD variants in 1Mb window) using GWAS summary statistics from glycemic traits^32^ and diverse T2D large GWAS meta-analyses^20,33–35^. Additionally, to further inspect the possible links between these loci and changes in gene expression, we annotated them with the summary statistics from expression quantitative trait loci (eQTL) in pancreatic islets^36^ and other tissues^37^, combined allelic specific expression (cASE) in islets^36^ and Variant Effect Predictor^38^. The results obtained were compared with a 1,000 randomization pairwise interaction control distribution.

The list of genes obtained for each pair from the annotations was analyzed to find any possible functional enrichment, mapping genes to their molecular functions, pathways, motifs, tissue specificity, and protein complexes^39^. The results obtained were compared with a 1,000 randomization pairwise interaction control distribution.

## Results

### Overall strategy

To develop, optimize and benchmark an MDR method to find variant interactions associated with T2D, we have used the Northwestern NuGENE project cohort^19^. The complete dataset is composed of 11,297,253 variants and 1,128 individuals. The data is stored in compressed CSV format, following the structure in Table 1. As we are studying the effect of variant interactions in disease risk development, we begin by analyzing pairwise combinations, the most basic interaction. In this dataset, the amount of possible pairwise combinations rises to approximately 6×10^13^. While MDR is a powerful method for detecting pairwise interactions, our need of analyzing the complete genomic dataset (billions of pairwise combinations) in a realistic amount of time and resources, forced us to reduce the input dataset. As a result, we applied a first filter to keep only the pairs of variants with a certain degree of association with the disease based on the results of a Chi-Square test *p*-value.

To deal with the huge volume of pairwise combinations we have used the MareNostrum IV and Greasy software^40^ to distribute more than 2,000 parallel tasks across 16 working nodes (48 CPUs and 96 GB of RAM), we have tested the association of the pairwise combinations of 11,297,253 variants in less than 4 days. Then, for the MDR, we have only kept the pairs with a degree of association with the disease (*p*-value<1×10^−6^), which corresponds to 1,883,192 pairs.

### Reduce the computational cost by leveraging High-Performance Computing technologies

In order to analyze the performance of the MDR and study the scalability, we have designed a virtual environment in OpenStack using 4 virtual interconnected nodes with 16 CPUs and 16GB of DDR3 RAM each. In the first phase of code optimization, we confirmed that the use of vector operations is key for the optimization, reducing the computational time by approximately 23 times: we pass from an average time of 28 minutes for every 1,000 variants to just 72 seconds.

In terms of scalability, our interest is in knowing how the application scales when more nodes are available and how it scales when more data is included. We have performed the following experiments:

#### Resources scalability

**Figure 2.**
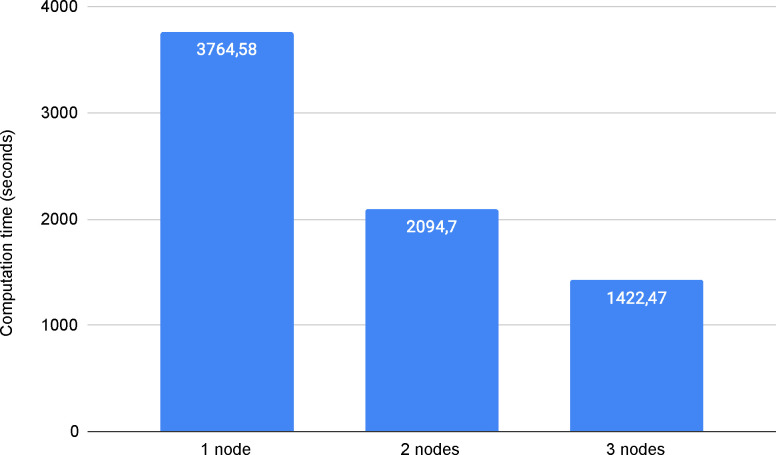
Computation time of processing 1,000,000 pair combinations using 1, 2, and 3 nodes.

We have processed 1,000,000 pair combinations in a distributed environment and tested with 1-node, 2-node, and 3-node as workers (Figure 2). The algorithm shows great scalability capabilities, reducing the computation time with the increase of working nodes. This is something to be expected since the computations are independent and we are using Apache Spark to distribute the work with HDFS for having the data distributed, achieving efficient communication across nodes, and avoiding unnecessary overheads that could affect the scalability capabilities.

#### Scalability problem

**Figure 3.**
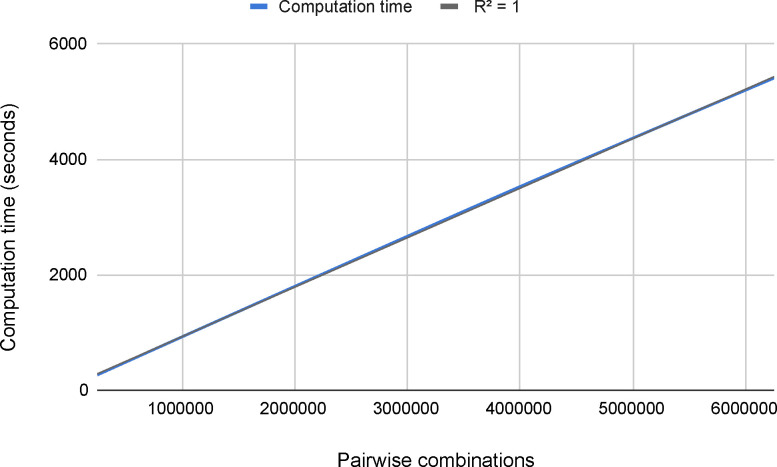
Computation time of processing different amounts of pair combinations using 3 working nodes.

To study the scalability of the problem, we have processed batches of data with different sizes (*N*=1M, 2M, 3M, 4M, 5M, 6M, Figure 3). As a result of this analysis, we observed that the behavior is completely linear (i.e., the execution time increases when the number of combinations increases in the same ratio). These results were expected since each pair is processed independently.

After testing the proposed methodology in an HPC environment, we were able to process 1 million combinations in 15 minutes using 3 regular machines. Importantly, despite the computational challenge that represents processing the 6×10^13^ possible combinations, the use of HPC technologies and the scalability of this application, will reduce the processing time to 100 days in a supercomputer such as Marenostrum IV, where there are 3,456 nodes with 48 cores.

**Figure 4.**
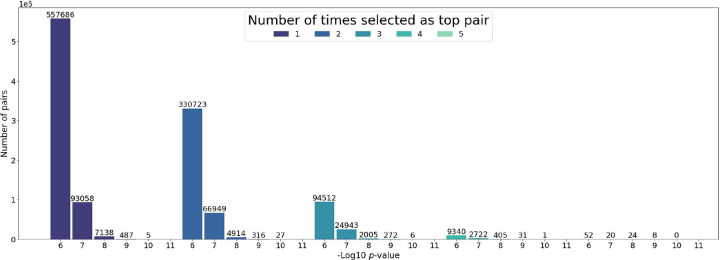
The *p*-value distribution in logarithmic scale for the studied pairs, grouped by the times they have been selected as top pairs from the 5 cross-validation datasets.

Figure 4 shows the distribution of the Chi-Square pairwise interaction *p*-values depending on the MDR consistency value, which corresponds to the number of times a pair appears at the top pairs of a cross-validation dataset. Consistently, the total number of pairs decreases after each step of the cross-validation, ending with only a few pairs that the MDR detects to be relevant independently of the dataset partition.

### Applying the MDR model to detect pairwise interactions associated with T2D

Using the same HPC environment with 3 working nodes, processing the 1,883,192 combinations takes no longer than 30 minutes (Figure 3). For selecting the final top pairs with the MDR, we have set a hard filter for keeping only the pairs that appear in 5 out of 5 CV sets as the top 20% prediction pairs. From the 1,883,192 pairs analyzed, we have obtained a set of 104 pairs (Supplementary Table S1). Importantly, 34 of these selected pairs have a *p*-value lower than 2.65×10^−8^ (5FDR with Bonferroni correction).

These results suggest that the discovery of the interacting pairs only depends on the calculated effect size that the variants will have with the disease, independently of how much lower the *p*-value is.

**Figure 5.**
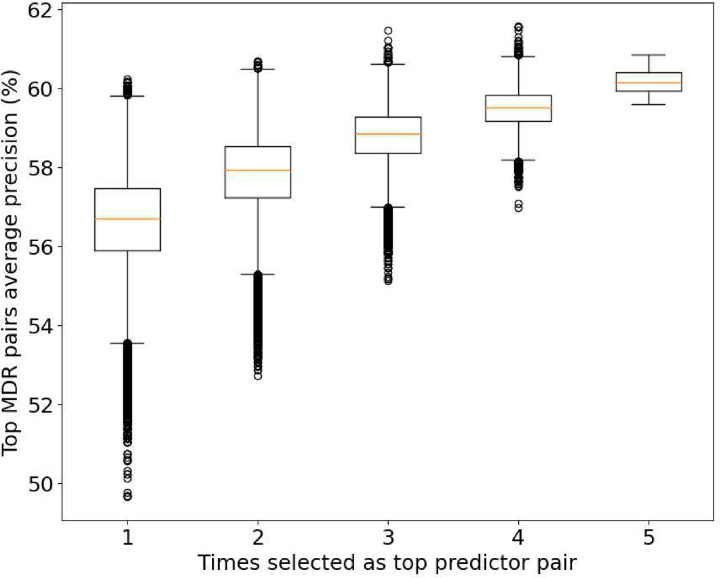
Box plot of the average prediction power (precision) for the studied pairs and their consistency value. To be selected as a top predictor in each CV set, a pair must be inside the 20% top predictor pairs of the set.

Regarding the significance level of the top 104 pairs, we have measured the prediction power of the top pairs from the MDR using the average precision (Figure 5) of the 5 CV sets. As expected, the group with the best average precision (60,17%) corresponds to the group of top pairs common across all the CV sets. However, some pairs have a higher average precision in other groups. This can be explained by overfitting in that set, meaning that its relation with the disease is completely related to the data analyzed.

### Functional interpretation of the T2D-associated pairs

**Figure 6.**
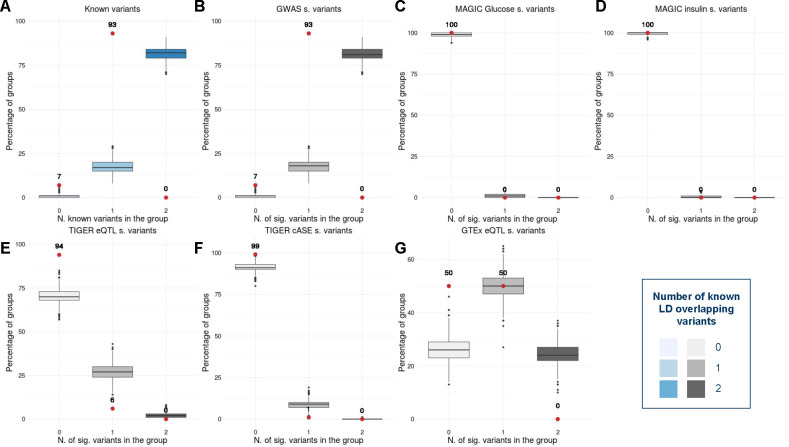
Box plots showing the percentage of already known variants in the interacting pairs in contrast with a control set in A) general, B) GWAS significant variants^20,33–35^, C) 2h glucose and fasting-glucose significant variants^32^, D) insulin significant variants^32^, E) pancreatic islets expression quantitative trait loci significant signals^36^, F) islets combined allelic specific expression significant signals^36^ and G) GTEx expression quantitative trait loci significant signals^37^. The red dots represent the percentage of variants in each pairwise interaction with a known annotation. The blue and grayscale box plots correspond to the distribution of the percentage of annotated variants in the control set.

In order to confirm the biological relevance of our findings, we annotated the variants and conducted a gene functional enrichment analysis. The 104 pairs of variants statistically associated with Type 2 Diabetes (T2D), obtained after applying the MDR method, contain 125 unique variants. Importantly, after annotating the 125 loci with GWAS meta-analyses and gene expression^20,32–37^, we observed that a large number of variants were already known when compared with a control set. More specifically, 93% (4.65-fold) of the variants are in LD with an already known T2D-related signal. These results, although expected, support our findings including the novel T2D-associations found by the MDR. Notably, 6% of the variants are in LD with an islet eQTL, 1% cASE, and 50% GTEx eQTL (Figure 6).

The list of genes obtained for each pair from these annotations, was used to conduct a gene functional enrichment analysis. Among the results obtained from the analysis, we found of particular interest the one derived from the pair composed of rs6450946 (chr5:33120048) and rs1219643 (chr10:123348355), which involves *FGFR2* signaling. Importantly, *FGFR2* is known to upregulate adiposity^41^. Decreased expression in this gene might be a compensatory response to enhanced adiposity, thus suggesting its possible relevance as a novel therapeutic approach for obesity^42^, and its overexpression has been suggested to be linked with embryo development and infertility in maternal diabetes^43^. In addition, the pair composed of rs200776585 (chr1:162626640) and rs72806227 (chr5:159084262) is connected with the regulation of hepatic stellate cell proliferation pathway. Interestingly, diabetic hepatic fibrosis is a progressive liver disease and a chronic complication of diabetes mellitus, which is known to be mainly caused by the activation of quiescent hepatic stellate cells (HSCs) by high glucose stimulation^44^. The activation of these cells is known to be promoted by *TSC22D4*^45^, a gene that acts as a critical controller of diabetic hyperglycemia^46^, and has elevated levels in patients with T2D with non-alcoholic fatty liver disease or steatohepatitis. In summary, these results suggest that the association found between the interactions and Type 2 Diabetes can be mediated by the connection between variants with gene expression and their effects on gene pathways.

## Discussion

Despite the fact that some studies have approached the analysis of variant interaction and its relation with complex disorders, it still represents a challenge. This is a consequence of the vast computational power needed to perform an extensive search across all the possible interactions at a genome-wide level. Thanks to technological advances, the combination of HPC methodologies with low-cost methods, such as the ones used in our study, is beginning to allow the analysis of the simplest interaction case: pairwise interactions. However, these low-cost methods have diverse limitations which can result in low statistical power to detect significant pairs in real genomic datasets^3^. In this study, we have confirmed that the use of more powerful methods, such as MDR, is key to detecting these significant pairwise interactions^47,48^.

However, even developing an optimized HPC architecture with some of the state-of-the-art technologies, processing a whole genomic dataset is still out of the picture, thus, limiting the analysis to a reduced dataset. For this reason, in this study, we have ensured some degree of association between the pairs and the disease using a Chi-Square method to filter and reduce the input dataset (*p*-value<1×10^−6^). Importantly, in contrast with other studies based on more strict filters^49,50^, and despite the reduction of the dataset to a feasible size, our proposed *p*-value threshold has facilitated the inclusion in the study of potentially significant pairs.

Furthermore, our implementation of the MDR to detect pairwise variant interactions has been implemented in a Singularity container, allowing the easy reproduction of the methodology in any environment. It is a plug-and-play framework, not needing to install any other software than Singularity itself, and being able to run on any operating system (OS). In comparison with traditional virtual machine environments, it also reduces the amount of resources needed, since no OS image is needed^29^.

Additionally, although it can be argued that other possible lines of work or hardware implementations may reduce the computational time, such as the use of GPUs or FPGAs^51^, these technologies are not typically available and may restrict the method access to other research groups. However, some interesting future work could focus on optimizing the method by further exploring different technologies.

The use of our MDR approach in a subset of 1,883,192 pairs has enhanced the discovery of 104 pairs of variants associated with Type 2 Diabetes (T2D). Importantly, although 93% of these associations correspond to already known T2D independent signals, 7% of them are novel and will need a further and detailed inspection. According to previous findings, two of the 104 pairs have some functional connection with the disease^41–46^, thus, suggesting gene expression and gene pathways as one of the putative underlying molecular mechanisms to mediate the link between these pairs and T2D and, therefore, to possibly enhance the development of the disease.

All these results place the proposed MDR architecture as a valid, efficient, and portable solution to study variant interaction in real reduced genomic datasets. Future work should focus on further optimization of the method to reduce the computational cost and be able to process bigger datasets. In this direction, some technologies could be explored, such as the implementation of a serverless architecture to make more efficient use of the available resources. Also, other implementations based on low-level code may be able to reduce the computational time but could impede portability. Finally, if we are able to increase the computational power of the method, we could begin to plan more complex experiments, such as across cohort studies or even higher-grade interactions, not being limited to pairwise combinations.

## Figures and Tables

**Table 1.** Three example rows of the input data. The first four columns contain the identification of the variant while the rest of the columns contain the value of the variant for each patient of the 1,128 individuals

chromosome	position	Ref allele	Alt allele	AA	Aa	aa	AA	Aa	aa	…	AA	Aa	aa

22	16231367	A	G	1	0	0	1	0	0		0	1	0
22	17052123	G	A	0	1	0	0	0	1		0	0	1
22	17055458	G	A	0	1	0	1	0	0		0	1	0

## Data Availability

The data that support the findings of this study are available from the Center for Genetic Medicine at Northwestern University but restrictions apply to the availability of these data, which were used under license for the current study, and so are not publicly available. Data are however available from the authors (gonzalo.gomez@upc.edu) upon reasonable request and with permission of the Center for Genetic Medicine at Northwestern University.
